# Orchestration of gene expression across the seasons: Hypothalamic gene expression in natural photoperiod throughout the year in the Siberian hamster

**DOI:** 10.1038/srep29689

**Published:** 2016-07-11

**Authors:** Ines Petri, Victoria Diedrich, Dana Wilson, José Fernández-Calleja, Annika Herwig, Stephan Steinlechner, Perry Barrett

**Affiliations:** 1 University of Veterinary Medicine Hannover, Buenteweg 17, 30559 Hannover, Germany; 2 Rowett Institute for Nutrition and Health, University of Aberdeen, Greenburn Road Bucksburn, Aberdeen AB21 9SB, UK; 3 Zoological Institute, University of Hamburg, Martin-Luther-King-Platz 3, D-20146 Hamburg, Germany

## Abstract

In nature Siberian hamsters utilize the decrement in day length following the summer solstice to implement physiological adaptations in anticipation of the forthcoming winter, but also exploit an intrinsic interval timer to initiate physiological recrudescence following the winter solstice. However, information is lacking on the temporal dynamics in natural photoperiod of photoperiodically regulated genes and their relationship to physiological adaptations. To address this, male Siberian hamsters born and maintained outdoors were sampled every month over the course of one year. As key elements of the response to photoperiod, thyroid hormone signalling components were assessed in the hypothalamus. From maximum around the summer solstice (late-June), *Dio2* expression rapidly declined in advance of physiological adaptations. This was followed by a rapid increase in *Mct8* expression (T3/T4 transport), peaking early-September before gradually declining to minimum expression by the following June. *Di*o3 showed a transient peak of expression beginning late-August. A recrudescence of testes and body mass occurred from mid-February, but *Dio2* expression remained low until late-April of the following year, converging with the time of year when responsiveness to short-day length is re-established. Other photoperiodically regulated genes show temporal regulation, but of note is a transient peak in *Gpr*50 around late-July.

The Siberian hamster (*Phodopus sungorus*) is a seasonal mammal and an exemplar for the pre-emptive physiological adaptations made by seasonal mammals to ensure survival and maximize reproductive success^1^,^2^,^3^. Physiological adaptations including body weight and regression of the reproductive axis, in preparation for winter are thought to be triggered by the predictable decrease in day length following the summer solstice, but recrudescence to the summer phenotype is orchestrated independently of day length (known as the photorefractory response) by an endogenous interval timer^4^,^5^,^6^. In continuous short days (SD), once recrudescence has taken place, the summer physiological state remains indefinitely if hamsters are not re-exposed to long-day (LD) photoperiod. Recrudescence requires 10–15 weeks of exposure to LD photoperiod or 4–6 weeks after the vernal equinox in simulated natural photoperiod, to initiate a SD physiological response or respond to melatonin infusions^7^,^8^. However, the mechanism involved in the development of refractoriness under SD conditions is unknown, as is the mechanism which terminates refractoriness and allows responsiveness to a SD melatonin signal.

From studies performed in both seasonal mammals and birds the current view of the mechanism governing seasonal phenotype centres on photoperiod regulated availability of thyroid hormone (T3) to the hypothalamus^9^. In LD, hypothalamic T3 availability is facilitated by TSH produced by the pars tuberalis (PT) acting on hypothalamic tanycytes to increase the expression of type 2 deiodinase (*Dio2*), enabling a higher rate of conversion of T4 to T3 to drive LD physiology. To enable SD adaptations, TSH expression is abolished which coupled with catabolism of T4 and T3 to inactive metabolites by SD induced type 3 deiodinase (*Dio3*) expression removes the thyroid hormone drive and initiates SD physiology^10^,^11^,^12^,^13^. In addition to deiodinase genes, other genes change expression in response to a change in photoperiod including the thyroid hormone transporter monocarboxylate transporter 8 (*Mct8*), intermediate filament proteins *Nestin* and *Vimentin*, components of retinol transport and metabolism, an orphan G-protein coupled receptor, *Gpr50* in tanycytes^14^,^15^, *Vgf* (non-acronymic) in the dorsal posterior ARC (dmpARC) and *Srif* (somatostatin) in the arcuate nucleus (ARC)^11^,^14^,^15^,^16^. However, the role of each of these genes in adapting physiology to the different seasons is still unknown.

Most studies investigating mechanistic basis of seasonal physiology are performed in a laboratory setting using static photoperiods representing long days (LD) or short days (SD), with hamsters abruptly transferred between static photoperiods to initiate physiological adaptations. While this approach has been instrumental in identifying the role of T3 and genes likely to contribute to the adaption of physiological states, static photoperiods do not reflect the natural environment where the hamster will experience a daily change in day length over the course of the year. The decremental changes following the summer solstice are relevant to registering photoperiodic history and have a strong synchronising effect on the recrudescence of reproductive physiology and body weight^5^,^8^,^17^,^18^. Therefore the aim of this study was to place gene expression changes into a temporal context in relation to body weight and reproductive physiology. The results reveal an orchestration of gene expression that may not necessarily be predicted from experiments performed with static photoperiods representing LD and SD and indicate that temporal organization will be important to seasonal physiological responses.

## Results

### Components of thyroid hormone metabolism and transport

The date of cull and the corresponding day length hours for hamsters born during March and April 2009 and culled between June and December 2009 are given in Table 1 and those born between March and July 2008 and culled between January and June 2009 are given in Table 2.

The body weight of a cohort of hamsters born during March and April ’09 representing the summer to winter transition and sacrificed on 21^st^ December as the final cohort, peaked at 10^th^ August (37.9 ± 1.6 g vs 26.9 ± 1.1 g at 18^th^ May) and declined thereafter, achieving significance at 12^th^ October (31.7 ± 1.6 g, P = 0.002; see Supplementary Fig. S1). Hamsters born between the months of March and July ‘08 entered the experiment at the beginning of January ’09 and represent the winter to summer transition. The representative cohort killed 16^th^ June ’09, started at 27.3 ± 0.3 g on the 6^th^ January and gradually increased, becoming significantly different by 10^th^ February (30.2 ± 1.2 g, P = 0.002, see Supplementary Fig. S1). Parallel changes in body weight, lean and fat tissue masses were evident in hamster cohorts used for analysis of hypothalamic gene expression (see Supplementary Fig. S1).

**Figure 1:**
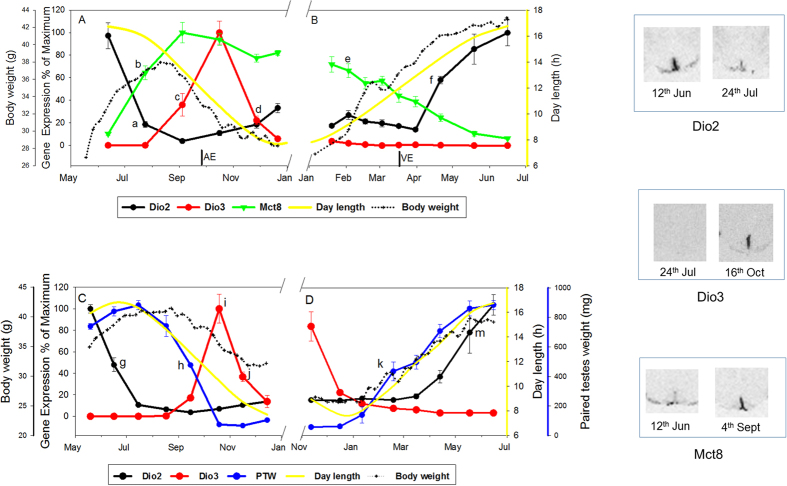
Temporal profile of gene expression for thyroid hormone signalling components, DIO2, DIO3 and MCT8 in the hypothalamus of Siberian hamsters in natural photoperiod over one year. Panel A: summer to winter transition and panel B winter to summer transition - experiment 1. In these panels, *Dio2* is represented by a solid black line; *Dio3* - red line; *Mct8* - green line; dotted black line - body weight change of the final cohort of hamsters born between March and April 2009 and killed 21^st^ December 2009 in panel A, and the final cohort of hamsters born March and July 2008 and killed 16^th^ June 2009 in panel B. ‘a’ P < 0.001 *Dio2* significantly decreased relative to 12^th^ June. ‘f’ P < 0.001 *Dio2* relative to 31^st^ March; ‘b’ P < 0.001 *Mct8* relative to June; ‘e’ P < 0.01 *Mct8* relative to a peak in August of the previous year; ‘c’ P < 0.001 *Dio3* relative to July; ‘d’ P < 0.001 *Dio3* relative to October. Panel C: summer to winter transition and panel D winter to summer transition - experiment 2. In these panels, *Dio2* is represented by a solid black line; *Dio3* - red line; paired testes weight – blue line; dotted black line - body weight change of the final cohort of hamsters born between 6^th^ February and 21^st^ March and killed 14^th^ December 2010 in panel C, and the final cohort of hamsters born between 1^st^ June and 3^rd^ July 2010 and killed 16^th^ June 2011 in panel D. ‘g’ P < 0.001 *Dio2* significantly decreased from 16th June relative to 12^th^ May; ‘m’ P < 0.001 *Dio2* significantly increased 18^th^ May relative to 13^th^ March. ‘i’ P < 0.001 *Dio3* relative to preceding time points; ‘j’ P < 0.01 *Dio3* significantly decreased relative to 19^th^ October. ‘h’ paired testes weight significantly decreased relative to 15^th^ July; ‘k’ P < 0.001 paired testes weight significantly increased relative to 11^th^ January. Superimposed on all plots (yellow line) is the actual day length over the months of the year for this transitional period (http://www.timeanddate.com/sun/germany/hannover). AE = autumnal equinox, VE = vernal equinox. N = 6–7 per group. Also Shown are representative autoradiograph images for *Dio2*, *Dio3* and *Mct8* at the dates indicated.

In early summer expression of *Dio2* in tanycytes was close to maximal value at 12^th^ June, declining sharply and were close to minimum by the 24^th^ July (18%, P < 0.001), remaining low for the remainder of the year (Fig. 1A). However, body mass continued to increase after July before declining from late August (Fig. 1A and see Supplementary Fig. S1). *Dio2* expression remained low until the 21^st^ April when expression increased (58%, P < 0.001) achieving 100% by 16^th^ June (Fig. 1B). Similar temporal kinetics were found in a second experiment (Fig. 1C,D). In the summer to winter transition *Dio2* was maximal at 19^th^ May, declining to 45% of maximum at the 16^th^ June (P < 0.001) and continued to decline thereafter. Similarly body and testicular mass continued to increase for several weeks after the 16^th^ June (Fig. 1C). In the winter to summer transition, *Dio2* expression remained suppressed until 13^th^ April when the first rise was observed (Fig. 1D).

*Dio3* expression in hypothalamic tanycytes was first detected at the 4^th^ September (36%, P = 0.002) with peak expression at 16^th^ October (P < 0.001), declining sharply by the 27^th^ November (22%, P < 0.001), undetectable by 21^st^ December (Fig. 1A) and during the following months up to 16^th^ June (Fig. 1B). Very similar temporal kinetics were found in a second experiment. In the summer to winter transition *Dio3* was undetectable until 15^th^ September, peaking at 19^th^ October (P < 0.001, Fig. 1C) and then declined sharply by 16^th^ November (P < 0.01). In the winter to summer transition *Dio3* was undetectable from December through to June (Fig. 1D).

*Mct8* expressed in hypothalamic tanycytes rose from a low expression (10%) at the 12^th^ June to 64% by July 24^th^ (P < 0.001) with the highest level at 4^th^ September (Fig. 1A). From September, *Mct8* gradually declined to almost complete absence by 16^th^ June, with the decline becoming significant at the 20^th^ January (P = 0.007, Fig. 1B).

### Non-thyroid hormone signalling genes

**Figure 2:**
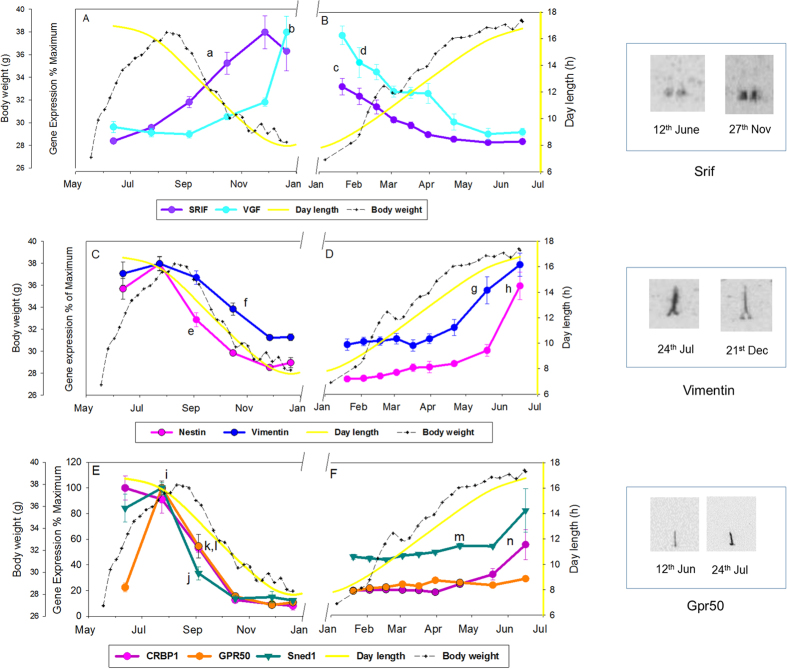
Temporal profile of neuronal or ependymal expressed genes in the hypothalamus of the Siberian hamster in natural photoperiod over 1 year. Panels A,C and E represent the summer to winter transition while panels B,D and F represent the winter to summer transition in experiment 1. Panels A and B: - *Srif* is represented by the purple line; *Vgf* – light blue. ‘a’ P < 0.001 *Srif* increased relative to 12^th^ June; ‘c’ P < 0.001 Srif decreased relative 27^th^ November of the previous year. ‘b’ P < 0.001 *Vgf* increased relative to 12^th^ June; ‘d’ P < 0.01 *Vgf* decreased relative to 21^st^ December of the previous year. Panels C and D: *Nestin* is represented by the pink line and *vimentin* - dark blue line. ‘e’ P < 0.001 *Nestin* decreased relative to 24^th^ July; ‘h’ *Nestin* increased relative 19^th^ May. ‘f’ P < 0.001 *Vimentin* decreased relative to 24^th^ July; ‘g’ P < 0.001 *Vimentin* relative to 21^st^ April. Panels E and F: *Gpr50* is represented by the orange line, *Sned 1* – dark green, *Crpb1* – dark pink. ‘i’ P < 0.001 *Gpr50* increased relative to 12^th^ June; ‘k’ P < 0.001 *Gpr50* decreased relative to 24^th^ July. ‘j’ *Sned 1* decreased relative 24^th^ July; ‘m’ P < 0.05 *Sned 1* relative to the minimal value achieved in December of the previous year. ‘l’ P < 0.001 *Crpb1* decreased relative to 24^th^ July; ‘n’ P < 0.001 *Crpb1* relative to the minimal value at the 31^st^ March. Superimposed on all plots (yellow line) is the actual day length over the months of the year for this transitional period (http://www.timeanddate.com/sun/germany/hannover). Dashed black line on plots represents the body weight change of the final cohort of hamsters born between March and April 2009 and killed 21^st^ December 2009 in panels A,C and E, and the final cohort of hamsters born March and July 2008 and killed 16^th^ June 2009 in panels B,D and F. N = 6–7 per group except April and May for Vgf where n = 5. Also shown are representative autoradiographs for *Srif*, *Vimentin* and *Gpr50*.

In experiment 1, *in situ* hybridization was used to investigate the expression of additional neuronal and ependymal expressed genes not involved in thyroid hormone signalling. In the first summer, *Srif* in the ARC gradually increased from 5% at 12^th^ June to 100% expression by 27^th^ November, with values achieving significance relative to June by 16^th^ October (P < 0.001, Fig. 2A). *Srif* subsequently declined and was significantly reduced compared to peak value by the 20^th^ January (P = 0.007, Fig. 2B) with expression continuing to decline gradually to less than 10% by 16^th^ June. A similar response was observed for *Vgf* expression in the dmpARC which gradually increased from 16^th^ October, becoming significant at the 21^st^ December (100% P < 0.001, Fig. 2A), then declining to less than 10%, achieving significance at 56% of maximum (P < 0.001) at the 3^rd^ February (Fig. 2B).

*Nestin* expression in hypothalamic tanycytes increased between 12^th^ June (77%) and 24^th^ July (100%, P = 0.033, Fig. 2C). By the 4^th^ September, *nestin* expression was reduced (49%, P < 0.001) and continued to decline thereafter (Fig. 2C). *Vimentin* in tanycytes showed a similar pattern, with maximal expression at the 24^th^ July declining to 34%, by the 16^th^ October (P < 0.001, Fig. 2C). Following the winter solstice, *Nestin* gradually increased but was not significant until 16^th^ June (71%, P < 0.001, Fig. 2D). *Vimentin* showed a similar pattern, starting at 32% at the 20^th^ January, gradually increasing, becoming significant relative to the 16^th^ March at 19^th^ May (67%, P < 0.001) and further increased by 16^th^ June (85%, Fig. 2D).

The highest level of cellular retinol binding protein (*Crbp1*) expression occurred at 12^th^ June, declined sharply by 16th October (12%), reaching significance at the 4^th^ September (P < 0.001, Fig. 2E) and remained low between January and April. Expression significantly increased at the 16^th^ June achieving 46% of maximum expression levels (P = 0.015 relative to 15^th^ May and earlier, Fig. 2F). Expression of the insulin responsive transcription factor, *Sned1* was maximal at the 24^th^ July and had declined sharply by 4^th^ September (33%, P < 0.001) and continued to decline to 12% of maximum (Fig. 2E). Following the winter solstice *Sned1* had a higher level of expression from the 20^th^ January becoming significant at 21^st^ April (P = 0.045), increasing to 77% expression by the 16^th^ June (Fig. 2F). *Gpr50* expression in tanycytes increased sharply to maximum expression levels between the 12^th^ June and 24^th^ July (100%, P < 0.001) and sharply declined by the 4^th^ September (54%, P < 0.001, Fig. 2E). *Gpr50* was not detected after the winter solstice at all time points up to 16^th^ June (Fig. 2F).

## Discussion

To date, the majority of studies of hypothalamic gene expression in response to photoperiod in any seasonal mammalian species have been performed by transferring animals abruptly between static long photoperiod (usually 16 h light: 8 h dark) and short photoperiod (usually 8 h light:16 h dark). In the Siberian hamster, this approach has successfully identified a substantial number of gene expression changes in several areas of the hypothalamus including the ARC, dmpARC and tanycytes^14^. Furthermore, only a few studies have investigated temporal changes in gene expression following a change in photoperiod, but again these studies have been performed by abruptly switching hamsters from LD to SD or acclimated hamsters in SD to LD^11^,^19^,^20^. Nevertheless, these studies revealed an intriguing temporal dependent expression of the mRNA for *Dio3*^11^. Together with experiments using hypothalamic microimplants releasing T3 into the hypothalamus these data provided evidence for a role of T3 in driving the LD physiological state, placing gene expression changes in thyroid hormone metabolism and transport at the centre of the seasonal regulatory mechanism^11^,^21^.

However, in their natural ecological domain, the hamster will experience an incremental increase or decrease in day length over the course of the year. These incremental changes in day length are relevant to registering photoperiodic history and have a strong synchronising effect on the recrudescence of reproductive physiology and body weight the following spring in juvenile hamsters born either side of the summer solstice^5^,^8^,^17^,^18^. Therefore aim of this study was to investigate the temporal kinetics of photoperiod regulated genes in a natural photoperiod over the period of one year in order to place hypothalamic gene expression, particularly genes involved in thyroid hormone signalling, into a temporal context in relation to body weight and reproductive physiology.

Although the current dogma would suggest elevated DIO2 is required to drive long day physiology, surprisingly the present data reveal a decline in *Dio2* expression can be initiated before day length shortens. Nevertheless, while *Dio2* declines there is a continued increase in body and testes mass indicating a programmed trajectory of growth, a notion supported by continued growth in juvenile hamsters following the large decrease in post-weaning *Dio2* expression^22^,^23^. One explanation for the temporal kinetics of *Dio2* expression in late spring/early summer after birth, is a decline programmed *in utero* or early post-natal development in these relatively young animals and would be consistent with evidence for a maternal influence of perceived photoperiod either in static or simulated natural photoperiod^17^,^24^. Although we were unable to quantify *TSHβ* expression in our hamsters due fragmentation and loss of PT tissue on removal of the brain or during sectioning of brain tissue, a second explanation for the decline in *Dio2* observed in hamsters during the first June after birth could be a LD refractory mechanism similar to that which occurs in sheep, which show a decline in PT derived *TSHβ* upon prolonged exposure to LD with a concurrent decrease in *Dio2* expression in the ependymal layer^25^,^26^.

Whether programmed *in utero* or part of a LD refractory mechanism, the decline in *Dio2* expression in LD hamsters offers an explanation for differences in responsiveness of *Dio2* to SD photoperiod seen in some studies which may simply reflect the extent to which *Dio2* expression has declined prior to SD exposure^11^,^14^.

*Dio2* expression remained suppressed following the winter solstice until mid-April while body mass and testicular recrudescence was evident in early February. This would suggest that a large increase in hypothalamic T3 is unlikely to be required to initiate the LD phenotype following the winter solstice.

However, the date of the first observed significant increase in *Dio2* expression may be highly relevant for the mechanism of breaking photorefractoriness. In studies using simulated natural photoperiod, hamsters have been shown to be refractory to SD until around 4–6 weeks after the vernal equinox at a durational day length of 13 h 49 min or greater^8^. At the 21^st^ April when *Dio2* mRNA was first measureable at 58% of maximum, the day length duration was 14 h 19 min, close to the duration of day length required to break photorefractoriness. Therefore we propose that a substantial rise in T3 would occur at this time and signals to break the photorefractory state. This would be consistent with studies in sheep where thyroidectomy prevents emergence from the photorefractory state^27^.

This finding has implications for the current dogma on the role of the PT from which LD induced TSH through a clock dependent mechanism of secretion is viewed as the driver of LD physiology^28^,^29^. While it is undisputed that TSH can induce *Dio2* expression in sheep and hamsters^12^,^13^, TSH is unlikely to be involved in the refractory response to SD since *Tshβ* is not produced in SD photorefractory hamsters or sheep^25^,^30^. A recent study in pinealectomized European hamsters held in LD over the course of one year did show a correlation between *Tshβ* expression in the PT and hypothalamic *Dio2* expression^31^ leading to the suggestion that a circannual rhythm of PT TSH secretion underpins the circannual rhythm in seasonal physiology. However, hamsters in that study were sampled when physiological adaptations had been established with a possibility that important temporal information prior to the changes had been missed.

The temporal kinetics of *Dio3* expression in natural photoperiod reflect the kinetics we have previously seen in hamsters transferred from static LD to SD^11^. Together, current and previous studies suggest a decline in photoperiod is the trigger for *Dio3* expression, but the transient expression profile of *Dio3* implicates an endogenous mechanism for the offset of expression. A candidate for this mechanism may involve DNA methylation on the *Dio3* promoter by photoperiod regulated DNA methyltransferase (DNMT3b) activity^32^. It is notable that the duration of day length after the summer solstice (16 h 48 min) at which *Dio3* increases (approximately 13 h 24 min) is convergent with the critical day length (13 h) determined to be required in static photoperiod experiments to initiate testicular regression^33^, suggesting the onset of *Dio3* expression is a principal event in the initiation of physiological adaptations.

During the decline of *Dio2* and before the peak of *Dio3* expression, *Mct8* expression increases to peak early September. This is coincident with the first increase in *Dio3* expression and the time at which body mass and testes start to decline. As DIO2 is a protein with a short half-life^34^, reduced levels of *Dio2* expression would lead to a relatively hypothyroid state in the hypothalamus. Therefore one explanation for the increase in *Mct8* expression would be to facilitate synthesis of more thyroid hormone transporter in an attempt to compensate for a reduction in DIO2 enzyme. However, limited evidence would suggest ependymal *Mct8* expression is unchanged or decreases in the hypothyroid state^35^,^36^. Given that a decline of T3 availability to the hypothalamus is a requirement for the induction of SD physiology, this inverse relationship of *Mct8* and *Dio2* is not consistent with this transporter maintaining the availability of T3 from the circulation or from the conversion of T4 to T3. Human MCT8 can both transport T3 and T4 in and out of a cell with the direction of transport is likely to depend on a concentration gradient between the extracellular and intracellular environment^37^. Human MCT8 has an approximately 3-fold higher affinity for T3 over T4^38^. If hamster MCT8 shows similar characteristics, it is likely that due to a 40–50 fold higher concentration of circulating T4 over T3 in the SD Siberian hamster^39^, T4 will most likely be the principal form of thyroid hormone transported by MCT8. This would be consistent with our hypothesis that this increase in *Mct8* contributes to a regulatory mechanism of thyroid hormone availability to the hypothalamus. This mechanism may involve the provision of T4 to enable substrate induced decrease in DIO2 protein half-life, or together with activation of *Dio3* expression, T4 conversion to rT3 which is inhibitory for DIO2 activity. Both actions would aid efficient and further depletion of hypothalamic concentrations of T3 by possible DIO2 protein synthesised from low residual *Dio2* mRNA^40^,^41^.

Based on a hypothesis that increased T4 transport via MCT8 augments a reduction in DIO2 activity and T3 generating capacity, the corollary of this is a decrease in MCT8 would lengthen the half-life of DIO2 deiodinase. Although we cannot rule out small increases in T3 produced by a small increase in *Dio2* expression which are below the limit of detection by autoradiography, we would propose the hypothesis that the remarkable gradual decline in *Mct8* expression seen from late autumn, coupled with a decrease in circulating T4^39^ permits a gradual increase T3 production. This would allow a gradual increase in body mass and reproductive recrudescence that is perfectly timed with the emergent environmental conditions of spring. Thus the date at which *Mct8* was detectably reduced below peak level may hold significance as it falls just ahead of the period when testicular recrudescence and body weight increases occur. This leads us to propose that gradual decline of *Mct8* may be instrumental in permitting the refractory response. Consistent with this is the previous observation of reduced *Mct8* expression in refractory hamsters in a continuous static SD experiment^14^. *Mct8* continues a decline in winter and spring and by the time of the first significant rise in *Dio2*, *Mct8* is almost at the lowest value, as might be expected to permit maximal T3 production.

*Srif* (somatostatin) has recently been shown to be up-regulated in the ARC in static SD and reduced by icv TSH indicating negative regulation by T3^13^,^22^,^42^. ARC derived somatostatin is proposed as a potential direct or indirect inhibitor of growth hormone secretion from pituitary somatotrophs^14^,^42^,^43^ and the kinetics of *Srif* expression observed in this study would be consistent with such a role, with maximal expression near the winter solstice when body weight is at the nadir, then through winter and spring of the following year showing a gradual decline. The similarity in the rate of *Srif* decline, as a gene negatively regulated by T3, parallels the decline of *Mct8* and would support the notion that this is reflective of a gradual increase in T3.

The role of *Crbp1*, *vimentin* and *nestin* in the context of the seasonal mammal is not yet clarified. However, a resurgence in *Dio2* expression occurred before significant increases in LD regulated genes such as *Crbp1*, *nestin*, *vimentin* and *Gpr50* and it would seem likely the role of these genes is downstream of T3 action.

*Sned1* is an insulin responsive transcription factor^44^, but regulated by T3 and photoperiod in tanycytes of rats and hamsters respectively^35^. LD hamsters have higher levels of circulating insulin in LD^42^; therefore the increase from January of *Sned1* may reflect increasing levels of circulating insulin as the LD phenotype develops after the winter solstice rather than an increase in T3.

In static photoperiods expression of *Gpr50* is higher in LD than SD photoperiods^15^ and might therefore be considered to be a LD expressed gene, possibly regulated by a LD induced TSH drive of PT origin or resultant T3 production. However, the temporal kinetics in natural photoperiod would suggest that *Gpr50* may not be regulated directly by TSH or T3 as *Gpr50* expression is ascendant as *Dio2* and photoperiod decreases, suggesting that this gene may be under the regulation of an intrinsic timing mechanism. A definitive function for *Gpr50* is lacking, but *Gpr50* knock-out mice are susceptible to food deprivation induced torpor^45^. The timing of this transient peak of *Gpr50* expression may hold significance for the timing of the onset of torpor in the hamster which occurs after 12 weeks of exposure in SD or around mid-December^46^. By June following the winter solstice, *Gpr50* expression only showed a small increase but this was not significant relative to the lowest value in January.

## Conclusions

Overall, the data illustrate the orchestration of gene expression in the hypothalamus of the Siberian hamster over the course of changing seasons. Strikingly there is a temporal restriction of components involved in thyroid hormone signalling including *Dio2*, *Dio3* and *Mct8*. While T3 can undoubtedly drive gene expression and downstream pathways resulting in LD physiology, the data suggest that once established, a high level expression of *Dio2* is not necessary for the maintenance of LD phenotype. Timing of maximal *Mct8* expression occurs as body mass and testes regress and is coincidental with the emergence of *Dio3* expression indicating that the temporal relationship of these three components may be necessary to achieve timely physiological adaptations. A resurgence of *Dio2* expression in late spring convergent with the time when photorefractoriness is broken, suggests that a large increase in hypothalamic T3 is not required to drive LD physiology but may be a signal to reset the sensitivity to melatonin.

## Materials and Methods

### Animals and tissue collection

Animal husbandry and all experiments were in accordance with the German Animal Welfare Act and approved by the Lower Saxony State Office for Consumer Protection and Food Safety. Djungarian hamsters (*Phodopus sungorus*) were reared under natural photoperiod and natural T_a_ (Hannover, Germany; ~52°N latitude). They were kept singly in polycarbonate cages (20.7 × 14 × 26.5 cm) and supplied with breeding diet (Altromin 7014) and tap water ad libitum, supplemented by a slice of apple once a week. Cages were provided with wood shavings and tissue for nest building. All animals were weighed weekly throughout the experiment.

Experiment 1 was carried out in two phases (timeline see Supplementary Fig. S1). For the winter-summer transition, 62 male hamsters were born under natural photoperiod and T_a_ from 30^th^ March to 16^th^ July 2008 and were killed every 2–4 weeks (N = 6–7) from 6^th^ January to 16th June 2009. Correspondingly, these animals had experienced one winter and were at the age of 9–13 months at the respective time point of killing. For the summer-winter transition, 42 male hamsters were born under natural photoperiod, between 16^th^ February and 22^nd^ April 2009. A cohort of seven hamsters were killed every 4–6 weeks between the 12^th^ June and 21^st^ December. They were 3–9 months of age when they were culled. All brains were stored at −80 °C until processed for coronal sections.

In the second experiment (timeline see Supplementary Fig. S2), for the autumn-spring transition group, 48 hamsters were born in natural photoperiod from 1st June to 3^rd^ July 2010 reared under natural photoperiod. From 12^th^ November 2010 a cohort of 6 hamsters were sacrificed at 4–5 week intervals resulting a one cohort per month from 12^th^ November 2010 to 16^th^ June 2011. For the summer-winter transition group 48 hamsters were born between 6^th^ February and 21^st^ March 2011. From the 19^th^ May one cohort of 6 hamsters were sacrificed every 4–5 weeks providing one cohort per month between 19^th^ May and 14^th^ December.

Hamsters were culled 3–4 hours after sunrise (www.timeanddatee.com/sun/germany/hannover). Hamsters were killed with carbon dioxide. Brains were immediately dissected, frozen on dry ice and then stored at −80 °C for later procedure of *in situ* hybridisation. Whole animal carcasses (minus the head) were sealed in plastic bags and also stored at −80 °C. Later, hamster carcasses sealed in their plastic bags were thawed and heated to 37 °C before being individually scanned by magnetic resonance imaging (MRI) (Echo MRI ™, Whole Body Composition Analyser, Echo Medical Systems, Houston, Texas).

### Riboprobes

Riboprobes complementary to fragments of the required DNA sequences were generated from Djungarian hamster, mouse or rat brain cDNAs by RT-PCR as described previously^11^,^15^,^16^,^39^,^47^,^48^,^49^. Templates for riboprobe synthesis were generated by PCR amplification of the insert from plasmid DNA using M13 forward and reverse primers which spans both insert and polymerase transcription binding and initiation sites in the host vectors. One hundred nanograms of PCR product was used in an *in vitro* transcription reaction with T7, T3 or SP6 polymerases as appropriate in the presence of ^35^S-uridine 5-triphosphate (Perkin-Elmer, Buckinghamshire, UK) for radioactive *in situ* hybridization.

### *In situ* hybridization

Coronal sections of the hypothalamic ARC region (14 μm thick) for each experiment were cut and analysed together. Sections were collected onto glass slides. Adjacent sections were mounted on consecutively numbered slides, permitting a number of mRNAs to be localised and quantified in each brain.

Briefly, frozen slides were fixed in 4% PFA in 0.1 m PBS, acetylated in 0.25% acetic anhydride in 0.1 m TEA, pH 8. Radioactive probes (approximately 10^6^ cpm) were applied to the slides in 70 μl hybridization buffer containing 0.3 M NaCl, 10 mM Tris-HCl (pH 8), 1 mM EDTA, 0.05% tRNA, 10 mM DTT, 0.02% Ficoll, 0.02% polyvinylpyrrolidone, 0.02% BSA, and 10% dextran sulfate. Hybridization was performed overnight at 58 °C. Following hybridisation, slides were washed in 4x SSC (1x SSC is 0.15 M NaCl, 15 mM sodium citrate), then treated with ribonuclease A (20 μg/μl) at 37 °C and finally washed in 0.1x SSC at 60 °C. Slides were dried and apposed to autoradiographic Biomax MR film (Kodak, Rochester, New York) for several hours to days.

### Image analysis

Films were scanned at 600 dpi on an Umax scanner and quantification was carried out using Image J 1.37v software (Wayne Rasband, National Institutes of Health, USA) or Image-Pro PLUS analysis software (v4.1.0.0, Media Cybernetics, Wokingham, USA). For each probe, three sections spanning a selected region of the hypothalamus were chosen for image analysis. Integrated optical density for each selected region was obtained by reference to a standard curve generated from the autoradiographic ^14^C microscale (Amersham). An average (with SEM) for the integrated optical densities for all sections of one animal and for all animals in one group was calculated. The highest value of one group in an assay was set to 100% expression value, and other treatment values were calculated accordingly.

### Statistics

The first detectable increase or decrease in body weight was determined using a two-way ANOVA, with main effect terms for time and animal ID followed by a Tukey post-hoc test.

All other statistical analysis was by one-way ANOVA followed by post-hoc Holm-Sidak test for multiple comparisons. In all cases the threshold for significance was P < 0.05.

## Additional Information

**How to cite this article**: Petri, I. *et al*. Orchestration of gene expression across the seasons: Hypothalamic gene expression in natural photoperiod throughout the year in the Siberian hamster. *Sci. Rep.* **6**, 29689; doi: 10.1038/srep29689 (2016).

## Supplementary Material

Supplementary Information

## Figures and Tables

**Table 1:** Summer to winter transition.

Date of cull (2009)	Day length (h:min)
12^th^ June	16:44
24^th^ July	15:55
4^th^ September	13:24
16^th^ October	10:36
27^th^ November	8:12
21^st^ December	7:40

Date of cull and corresponding day length experienced by Siberian hamsters born between March and April 2009 and housed outdoors in natural photoperiod. Note the summer solstice day length duration on the 21^st^ June was 16 h:48 min.

**Table 2:** Winter to summer transition.

Date of Cull (2009)	Day length (h:min)
20^th^ January	8:27
3^rd^ February	9:12
17^th^ February	10:05
3^rd^ March	11:01
17^th^ March	11:58
31^st^ March	12:55
21^st^ April	14:19
19^th^ May	15:56
16^th^ June	16:47

Date of cull and corresponding day length experienced by Siberian hamsters born between March and April 2008 housed outdoors in natural photoperiod.
