# Supplementary material for: Orchestration of gene expression across the seasons: Hypothalamic gene expression in natural photoperiod throughout the year in the Siberian hamster
Source: Sci Rep. 2016 Jul 11;6:29689. doi: 10.1038/srep29689 (PMC4942572; doi:10.1038/srep29689)
Supplement: Supplementary Information [file srep29689-s1.pdf]

SUPPLEMENATARY INFORMATION

Orchestration of gene expression across the seasons: Hypothalamic gene expression in natural photoperiod over a year in the Siberian hamster

Ines Petri, Victoria Diedrich, Dana Wilson, José Fernández-Calleja, Annika Herwig, Stephan Steinlechner and Perry Barrett

Supplementary figure legends

Supplementary Fig. S1 Experimental timeline and body mass data for Siberian hamsters of the course of one year (experiment 1). Green bar indicates period over which hamsters were born; brown bar – the period hamsters were maintained prior to start of killing for tissue collection; blue bar – period over which hamsters were killed for tissue collection; (A) Body weights of hamsters born in March and April 2009 and killed on the 21st December representative of the transition from summer to winter; (B) Body mass of each cohort of hamsters at the time of killing; (C) Lean mass and (D) fat mass determined by Echo MRI of cohorts at each time point. (E) Representative of the transition from winter to summer, body mass of hamsters born between March and July 2008 and killed 16th June 2009. (F) Body mass of each cohort of hamsters at the time of killing; (G) Lean mass and (H) fat mass determined by Echo MRI of cohorts at each time point. Numbers in A and E indicate time points cohorts of hamsters from the study were killed for the assessment of hypothalamic gene expression. 'a' indicates the date (21st June) the first significant increase in body mass relative to start at 18th May ($P<0.001$); 'b' first significant decrease in body mass relative to maximum at the 10th August ($P=0.002$); 'c, d, e' significant decrease in body mass ($P=0.002$), lean ($P=0.003$) and fat mass ($P<0.001$) respectively of individual groups used for collection of tissue relative to peak value at 24th July; 'f' indicates first significant increase in body mass relative to start at 7th January ($P=0.002$); 'g, h, i' significant increase in body mass ($P=0.009$), lean ($P=0.021$) and fat mass ($P=0.006$) respectively of individual groups used for collection of tissue relative to 7th January. N=6-7 per group.

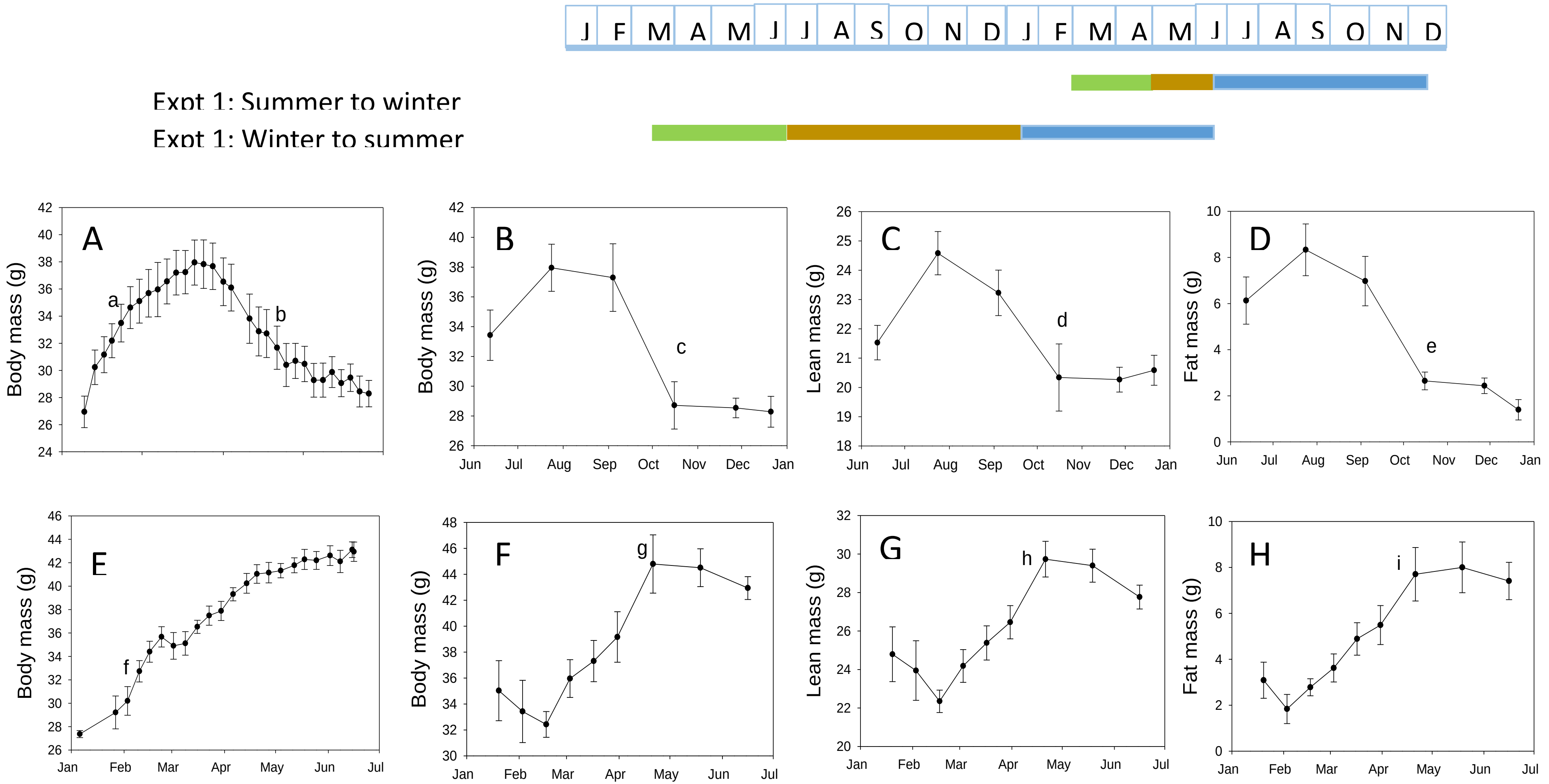
J F M A M J J A S O N D J F M A M J J A S O N D
Expt 1: Summer to winter
Expt 1: Winter to summer
A
Body mass (g)
a
b
B
Body mass (g)
c
C
Lean mass (g)
d
D
Fat mass (g)
e
Jun Jul Aug Sep Oct Nov Dec Jan
E
Body mass (g)
f
F
Body mass (g)
g
G
Lean mass (g)
h
H
Fat mass (g)
i
Jan Feb Mar Apr May Jun Jul

Supplementary Fig. S2 Experimental timeline and body mass data for Siberian hamsters over the course of one year (experiment 2). Green bar indicates period over which hamsters were born; brown bar – the period hamsters were maintained prior to start of killing for tissue collection; blue bar – period over which hamsters were killed for tissue collection. (A) Body mass of the final cohort of hamsters born in spring 2010 representing the change in body mass across the transition from summer to winter 2010; 'a' indicates the date (21st June) when body mass was significantly greater relative to bass mass at 18th May ($P<0.001$); 'b' indicates date (12th October) at which body mass was significantly decreased relative to the maximal body mass at 31st August ($P=0.002$). (B) Body mass of the final cohort of hamsters born in spring 2010 representing the change in body mass across the transition from winter to summer 2011. 'c' indicates the date (10th Feb) at which body mass was significantly increased relative to 5th January ($P=0.002$). $N=6$ for all groups.

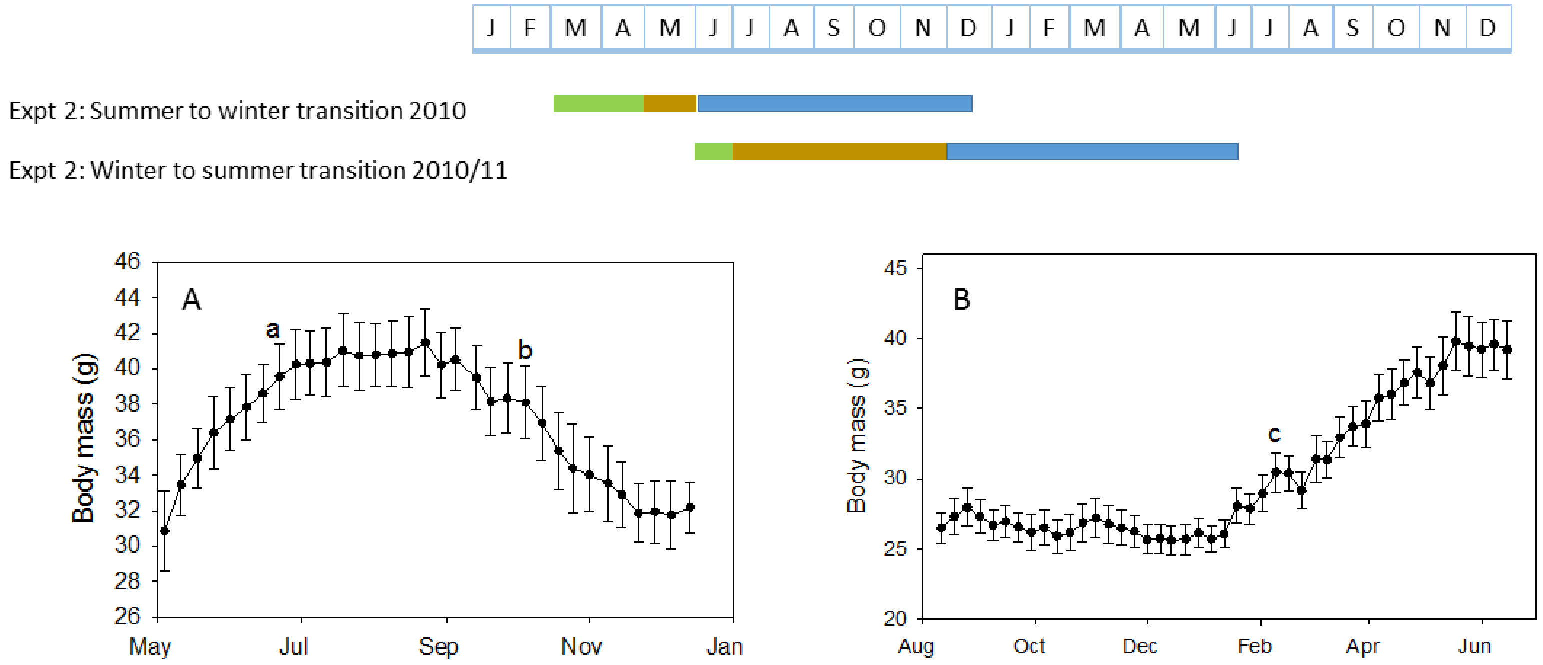
J F M A M J J A S O N D J F M A M J J A S O N D
Expt 2: Summer to winter transition 2010
Expt 2: Winter to summer transition 2010/11
A
Body mass (g)
46 44 42 40 38 36 34 32 30 28 26
a
b
May Jul Sep Nov Jan
B
Body mass (g)
45 40 35 30 25 20
c
Aug Oct Dec Feb Apr Jun
